# Robot Guided ‘Pen Skill’ Training in Children with Motor Difficulties

**DOI:** 10.1371/journal.pone.0151354

**Published:** 2016-03-11

**Authors:** Katy A. Shire, Liam J. B. Hill, Winona Snapp-Childs, Geoffrey P. Bingham, Georgios K. Kountouriotis, Sally Barber, Mark Mon-Williams

**Affiliations:** 1 School of Psychology, University of Leeds, Leeds, United Kingdom; 2 Bradford Institute of Health Research, Bradford Teaching Hospitals NHS Trust, Bradford, United Kingdom; 3 Department of Psychological and Brain Sciences, Indiana University, Indiana, United States of America; 4 Department of Psychology, Manchester Metropolitan University, Manchester, United Kingdom; Tokai University, JAPAN

## Abstract

Motor deficits are linked to a range of negative physical, social and academic consequences. Haptic robotic interventions, based on the principles of sensorimotor learning, have been shown previously to help children with motor problems learn new movements. We therefore examined whether the training benefits of a robotic system would generalise to a standardised test of ‘pen-skills’, assessed using objective kinematic measures [via the Clinical Kinematic Assessment Tool, CKAT]. A counterbalanced, cross-over design was used in a group of 51 children (37 male, aged 5–11 years) with manual control difficulties. Improved performance on a novel task using the robotic device could be attributed to the intervention but there was no evidence of generalisation to any of the CKAT tasks. The robotic system appears to have the potential to support motor learning, with the technology affording numerous advantages. However, the training regime may need to target particular manual skills (e.g. letter formation) in order to obtain clinically significant improvements in specific skills such as handwriting.

## Introduction

It is consistently found that motor skill ability predicts later academic attainment in both reading and mathematics [1–4], and that difficulties with motor skill leads to a range of negative physical, social and mental consequences [5]. There is good evidence that the negative outcomes associated with motor impairment can be minimised if identified early enough and treated with appropriate intervention [6–8], yet there is currently no clear agreement on the most effective method for intervention [9]. One intervention strategy that is showing increasing promise in the field of rehabilitation medicine is the use of robotic systems to support motor skill acquisition, based on sound and well-established principals of sensorimotor learning [10,11]. Robotic devices can constrain the ‘action space’ that needs to be explored by a user when learning a movement pattern [12–16]. This can allow actions to be more accurate from the start, enabling the creation of appropriate internal models, which can be refined more effectively using tailored assistive forces (e.g. increasing resistance when the user moves away from a defined spatial path). Whilst too much assistance may actually hinder learning of movements for healthy individuals, for individuals with disabilities or difficulties with movement, these systems do show potential [12–16]. Moreover, robotic interventions have a number of advantages over traditional approaches. The ‘dosage’ of the intervention can be strictly controlled, meaning each child can receive the same therapeutic experience, a single supervisor can provide guided treatment to a group of children at once, rather than on a one-to-one basis, and therapy can be embedded within computer games that children enjoy, find motivating and are non-stigmatising (with potential concurrent benefits of improved self-efficacy).

On this basis, a robotic intervention system for training manual dexterity was designed with the potential to be used within a school or home environment without the need for one-to-one administration [17,18]. Users interfaced with the system by grasping a stylus attached to a haptic robotic arm and moved the arm to complete a series of trials within a ‘computer game’ that required them to move the stylus along a three dimensional (3D) path. The robotic arm assisted the user in making the correct movements by providing resistive forces when the stylus left a defined path, whilst still requiring active prospective control from the participants. This type of control, rather than passive guidance, has been found to be necessary for effective learning [19].

Initial studies investigating the feasibility of using this system demonstrated that it can ameliorate differences in performance between children with and without clinical movement difficulties [17], of different ages [20], and between children dichotomously classified as either ‘high’ or ‘low’ for their score on a visual perception [VP] test [18]. Moreover, there was some evidence for generalisation of the robotic system in the study of children with differing VP status (i.e. training benefits accruing on a different task). Children in both groups were found to have performed better on a digital tablet-based drawing task post-haptic training. This raises the possibility that the system could be used to target a critical daily activity for children within schools—handwriting. There is an urgent need for cost-effective solutions to support children with handwriting difficulties within schools and therefore a possible exciting application of the system.

The current study therefore set out to investigate the generalisability of training benefits to a previously established measure of manual ‘pen-skills’; namely the Clinical Kinematic Assessment Tool [CKAT] [21]. The CKAT uses the same underlying software and equipment as the drawing task used in previous studies [18,20] and provides detailed kinematic feedback on performance comparable to laboratory motion capture systems [21]. The battery is designed to tap into many of the skills required in other ‘real-life’ manual tasks such as handwriting (e.g. exerting precise forces on a stylus and making use of feedforward and feedback control). The CKAT has previously been used in a number of studies with children in order to assess motor skill and has been shown to be capable of distinguishing between children’s levels of motor coordination on the basis of their functional ability, age and gender [22,23]. It therefore makes an ideal assessment to test whether training benefits are able to generalise to the manual skills that directly underpin handwriting.

The present study utilised a counterbalanced crossover design that investigated haptic-training effects in children identified as having manual coordination difficulties. Consequently, for the first time, it is was possible to directly investigate whether: (i) post-training benefits on the novel robotic arm tasks were directly attributable to the training rather than being a function of natural improvement due to practice effects or baseline ability; (ii) there was evidence of generalisation of the benefits on the CKAT battery of manual control; (iii) any benefits observed in performance were sustained even after training was withdrawn.

## Method

### Participants

All the children in the study attended a local primary school in the city of Bradford (West Yorkshire, UK) with 470 children on the school roll for 2013–14. The class teachers put forward those children they believed had ‘handwriting difficulties’ (n = 101), which represented 21% of the whole school. These children subsequently were tested using the manual dexterity section of the Movement Assessment Battery for Children 2^nd^ Edition [24], the most commonly used standardised test of motor ability in Europe [9]. Fifty nine of these children (12.5% of the school population) scored under the 15^th^ percentile on the manual dexterity section, indicating risk of manual coordination deficits [24]. Informed written consent was obtained from the Head-teacher of the participating school (acting in loco parentis for their students). The school also obtained informed consent internally from the parents/guardians of children the school identified as having handwriting difficulties, giving them advanced notice of the study and their right to opt out should they wish to do so. Written information detailing the study was sent out to all families and they were given the opportunity to discuss the study verbally with informed staff members and the research team if they had further questions. Parents/guardians wishing to withdraw (opt-out) were able to do so either verbally or in writing (both recorded by an in-school coordinator). These multiple response methods and the embedding of this process in school, with the support of trusted staff-members, ensured that all parents/guardians were engaged with the consent process and that they had multiple modes by which they could let their wishes be known. Ultimately, consent to take part in the objective screening for manual difficulties was obtained for 101 children, with 51 of these children (see Table 1) subsequently meeting the inclusion criteria for participation in the trial of the robotic intervention. Children gave their verbal consent immediately prior to their participation in each phase of the study (i.e. consented for screening and then again for participating in the trial) after having the study explained to them. Verbal consent was used for children since not all children in this age range could provide written consent. The University of Leeds Ethics and Research committee approved these consent procedures and all other aspects of the study’s design and methodology, ref no: 13–0112, date approved: 23-Jul-2013.

**Table 1:** Descriptive statistics of sample, split by counterbalance group.

		Age (in years)	Gender		Handedness		MABC-2 MD (%ile)	MABC-2 MD <5^th^ percentile
Counterbalance Group	n	Mean [Range]	Male	Female	Right	Left	M (SD)	n
A	26	8.15 [5.10–10.96]	20	6	23	3	5.46 (3.31)	16
B	25	8.29 [5.29–10.92]	14	11	24	1	4.58 (3.77)	16
Total	51	8.22 [5.10–10.96]	34	17	47	4	5.03 (3.53)	32

For whole sample, and split by counterbalance group: age at initial baseline testing, gender, handedness, percentile score on Movement Assessment Battery for Children(Second Edition) manual dexterity subsection [MABC-2 MD], and number of children scoring under 5^th^ percentile on MABC-2 MD subsection. A = Counterbalance group receiving Haptic-Training in time period 1 but not 2; B = Counterbalance group receiving Haptic-Training in time period 2 but not 1

### Materials

#### Haptic-training

The robotic system used in this experiment was the same as that used in previous studies [17–19] and consisted of a stylus attached to a haptic device: the PHANTOM Omni (Sensable Technologies, Inc.). The PHANTOM is an impedance control device which outputs a force in reaction to the user moving the input device (stylus) to interact with a virtual 3-D environment displayed on a computer screen (as a 2.5D image–i.e. with pictorial cues providing an impression of depth). The force used for this intervention was modelled as a virtual spring, which pulled the stylus back onto the nearest point on a correct spatial path when participants showed spatial deviation from it. The stiffness of the spring could be altered in order to adjust task difficulty, with the force set at six different levels of difficulty corresponding to forces of 2.02N, 1.08N, 0.83N, 0.57N, 0.35N and 0.13N. The virtual spring length was set at 0.5cm from the centre of the movement path. If the stylus was located within this threshold distance there were no forces applied. This did not involve any actively applied forces (as it was an impedance rather than admittance controlled device), and there was therefore no safety concerns to the children.

**Fig 1:**
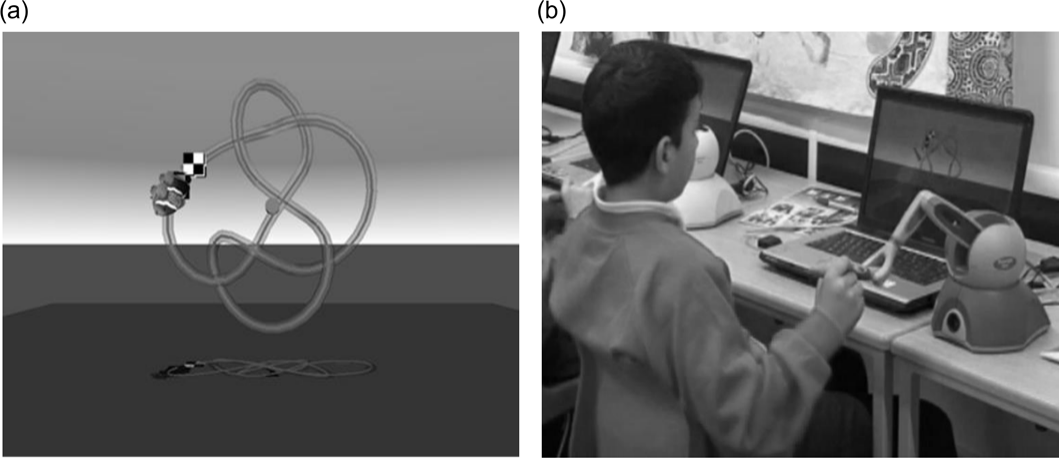
Set-up of the robotic arm system. The child traces around a 3-D path represented on the laptop screen (a), using the pen attached to the robotic device (b).

The trials presented during haptic-training were the same as those used in previous studies and involved using the stylus to push a fish icon around a 3-D movement path shaped like a three-dimensional knot (see Fig 1A). Participants began at a fixed starting location and moved their icon to a finishing location, whilst racing against computer-controlled ‘competitor’ fish presented on a 15 inch diameter laptop screen (Fig 1B). The paths themselves varied in length, curvature and torsion. At the beginning of a training session all children were given two practice trials, consisting of a circle and a loop, in order to familiarise them with the task requirements. After this practice all the children were able to complete the paths on the highest (easiest) level of assistance (i.e. greatest stiffness).

**Fig 2:**
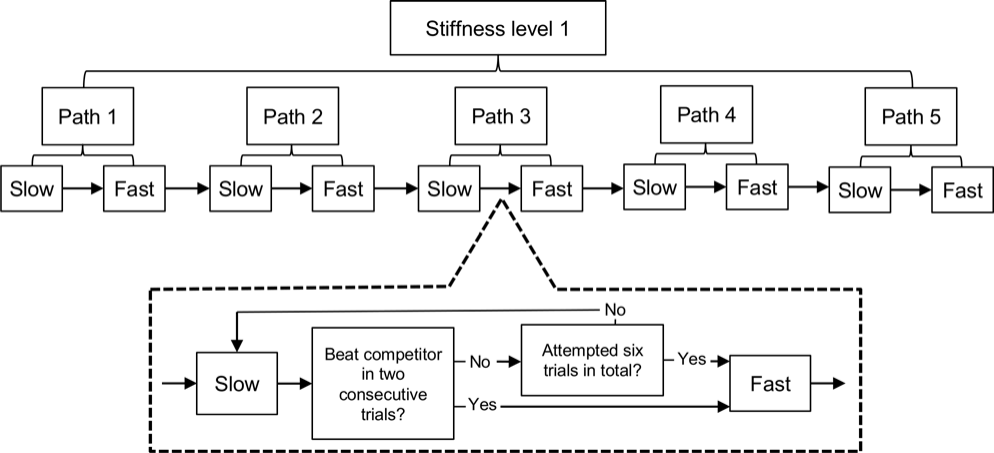
Schematic of training blocks. Child progresses through slow, then fast conditions for each of the five paths in a training block. Once completed, stiffness level is reduced for the next block.

For training purposes six blocks of trials were created, one to correspond with each of the stiffness levels that participants were trained on (i.e. haptic-assistance across all trials within a block was constant). Within all blocks the same sequence of trials was presented, which comprised of training on five unique paths (numbered 1 to 5) always presented consecutively in the same fixed order and with the complexity of the path increasing from Path 1 to Path 5. Within a block, task difficulty between trials was further manipulated by altering the speed of the competitor fish, with it taking 25 seconds to complete the path in a ‘slow’ condition trial (starting when the child successfully placed the stylus on the ‘start’ square), and 15 seconds in a ‘fast’ condition trial. Children started on the ‘slow’ condition on Path 1 and had to beat the competitor fish twice in a row, by reaching the end of the path ahead of it (with a maximum of six attempts). They then completed the same path in the ‘fast’ condition. Once they had completed these trials, they progressed to the ‘slow’ condition on path 2 and so on. A full block constituted completing all five paths at both race speeds (Fig 2). In order to reduce frustration, if the child did not complete a path in 90 seconds after they first reached the start square, the trial was terminated.

#### Outcome measures

Participants completed the following battery of assessments before and after haptic-training and a control period:

#### Novel haptic-system trials

Using the same haptic system as deployed during training (i.e. Fig 1) participants were tested on a single novel path (not presented during training) across twelve consecutive trials. This sequence of trials started at the highest assistance level for two trials then proceeded through a further pair of trials at each assistance level in descending order. If the participant did not complete a trial in 90 seconds after they first reached the start square, the trial was terminated and they proceeded automatically to the next one in the sequence. If children failed to complete a trial then a maximum score of 90 seconds was awarded instead. The outcome variable for these trials was the median time (in seconds) to complete the paths. Other measures, such as path length, which were recorded in previous studies were found to merely reflect this variable [17] and so they were not used as an outcome measure in this study.

#### Clinical Kinematic Assessment Tool [CKAT]

The primary outcome measure for this study was an objective measurement of pen skills. In order to assess pen skills, participants were tested on the Clinical Kinematic Assessment Tool (CKAT) [21,23]. The CKAT test battery consisted of three tasks (tracking a moving dot, aiming between a series of dots and tracing along a path), all performed with a ‘pen’ stylus on a tablet computer. The CKAT collects a large number of kinematic variables. However, for this study, only one outcome measure for each task was investigated as these measures have previously been shown to be sensitive to manipulations in task-difficulty, age and gender in 4 to 11 year olds [23]. For the tracking task, average root mean square error was calculated as the straight-line distance (in mm) between the centre of the target and the tip of the stylus at each sampled time point. For the aiming task, movement time in seconds (MT) was calculated by taking the median MT for each of the aiming movements. For the tracing task, a penalised path accuracy score was calculated, by inflating path accuracy (the mean distance away from an idealised reference path in mm) by the percentage deviation from the optimal movement time of thirty-six seconds, to give a composite measure of performance reflecting both the speed and accuracy demands of this specific task.

The US authors took the opportunity to take additional measures of the drawing ability of the children (to replicate their previous work [20]) but these measurements were not part of the primary study and have not been reported (or considered) within this manuscript.

### Procedure

A crossover design was used in this study, with children being split into two groups (A and B), with allocation counterbalanced for age (see Table 1 for descriptive statistics of participants). Two time periods of 5-weeks were split by a 3-week gap (due to school holidays). Participants were assessed at the beginning and end of each time period on the battery of outcome measures already described. Group A received haptic-training during the first time period whilst Group B received no intervention. Group B then received the intervention in the second time period, whilst group A received no intervention. This allowed the natural improvement of the children over time on the outcome measures to be taken into account when investigating additional, training specific, effects.

The intervention followed exactly the same procedure as that used in previous studies assessing the system [17–20]. During the intervention period in which participants received haptic training, participants took part in one 20 minute long session per week. Children were brought out of class in groups of five to sit in a quiet, otherwise unused classroom, under the supervision of two researchers. The robotic arm systems were set up on tables next to each other, at height-appropriate tables. The laptop screen was set up directly in front of them, with the robotic arm placed on the right of the computer (or the left for the left-handed children). The children were allowed to progress at their own rate through the training blocks, which were delivered in a sequential order starting with the block providing the greatest assistance (i.e. highest stiffness). After each session, if they had progressed more than halfway through the current training block, they would start the next session at the beginning of the next block. If not, they started on the same block again.

### Analysis

The two primary independent variables under investigation in this study were: participant’s counterbalance group (A or B), and time of assessment (baseline and post-test for intervention period 1 and 2). Due to the skewness and kurtosis of the outcome measure scores for the Robotic Arm novel task and the CKAT tasks, the outcome measures were reciprocally transformed, enabling parametric tests to be used. Dependent variables for each task were analysed separately using 2x4 mixed ANOVAs that specified counterbalance group as a between-subjects factor (Group A or Group B) and time of assessment as a within-subjects factor (baseline 1 [B1], post-test 1 [P1], baseline 2 [B2] or post-test 2 [P2]). Some exploratory analysis was then done, splitting the participants by age and manual coordination ability, in order to look in greater depth at the potential influence of these additional factors. Such analysis also allowed for more detailed consideration of the feasibility of using the intervention equipment with younger children than tested in previous studies [17,18].

## Results

All the children reported that they enjoyed taking part in the training and were able to complete the tasks, with only one child withdrawing from the study (due to leaving the country). There were some missing data due to recording errors; participants needed to have valid data at all four time points in order to be included in the analysis for each test. Table 2 gives descriptive statistics for each of the time points for intervention groups A and B, and total valid data for each test. There was an issue with extreme outliers within the data on the Robotic Arm, CKAT tracing and CKAT aiming tasks (|*z*| > 2.5), and so analyses were run with and without these cases included.

**Table 2:** Descriptive statistics of outcome measures at each time point split by intervention group.

		Baseline 1	Post-test 1	Baseline 2	Post-test 2
	Total valid n	Mean[range]	Mean[range]	Mean[range]	Mean[range]
Robotic Arm^1^					
A	20	32.66 [10.31, 80.01]	9.63 [6.32, 17.43]	8.39 [4.82, 15.38]	7.89 [5.80, 11.00]
B	21	35.15 [17.28, 71.07]	26.70 [10.23, 84.41]	16.04 [9.47, 34.03]	8.20 [4.94, 19.03]
C-KAT: Tracking^2^					
A	24	15.06 [8.34, 36.59]	13.70 [8.77, 27.80]	13.32 [7.57, 26.30]	14.17 [8.36, 32.65]
B	21	17.26 [9.09, 51.19]	17.48 [9.40, 43.76]	17.01 [8.45, 66.74]	18.71 [8.69, 40.66]
C-KAT: Aiming^3^					
A	13	1.70 [1.15, 2.64]	1.65 [1.20, 2.13]	1.52 [1.07, 2.30]	1.62 [1.11, 2.26]
B	10	1.59 [1.10, 2.04]	1.55 [1.23, 2.18]	1.43 [1.17, 2.47]	1.44 [1.20, 1.78]
C-KAT: Tracing^4^					
A	23	1.58 [0.87, 2.63]	1.46 [1.02, 2.48]	1.42 [1.06, 1.96]	1.40 [0.86, 2.25]
B	24	1.79 [1.09, 5.62]	1.58 [1.01, 3.19]	1.84 [0.96, 4.50]	1.63 [0.91, 4.59]

Scores shown are pre-transformation and with all outliers included

^1^ Robotic Arm scores: median time (in seconds) to navigate paths

^2^ C-KAT tracking measure: root mean square error

^3^ Aiming measure: average movement time

^4^ Tracing measure: penalised path accuracy

### Preliminary data exploration

Initial checks using independent t-tests were carried out to ensure that baseline scores on the outcome measures did not vary between the two intervention groups. Scores on the robotic arm novel task did not vary significantly t(1, 47) = 1.30, *p* = .200, and neither did scores on the CKAT tasks: tracking t(1, 48) = .27, *p* = .788; aiming t(1, 40) = 0.11, *p* =. 910; and tracing t(1, 49) = 0.43, *p* = .668. In addition, there was no significant difference between groups regarding their performance on the MABC screening assessment t(1, 49) = 0.90, *p* = .374.

### Robotic Arm novel task

**Fig 3:**
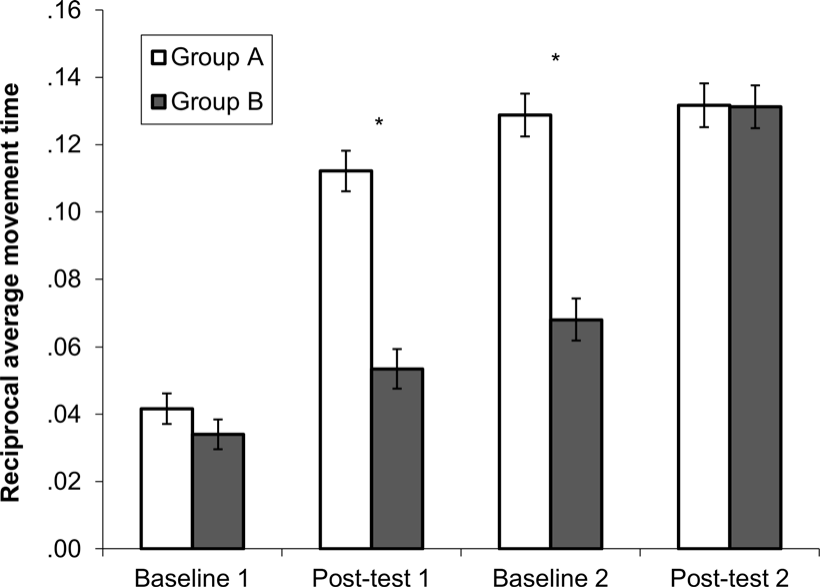
Task performance on novel robotic arm task. *p < .05, **p < .001 Reciprocal average time (in seconds) to complete all paths on novel robotic arm task (with standard error bars) for each group at each time point.

We firstly examined the impact of the intervention on the novel task conducted on the robotic arm system. A mixed 2x 4 ANOVA was conducted, with intervention group as a between subjects factor (Group A (received intervention in Time 1) or Group B (received intervention in Time 2)), and time period entered as the within subject factor (baseline 1 [B1], post-test 1 [P1], baseline 2 [B2], post-test 2 [P2]). With the outliers included, there was a significant main effect of time F(3, 117) = 119.10, *p* < .001, ηp^2^ = .75 and group F(1, 39) = 33.79, *p* < .001, ηp^2^ = .46, as well as a significant interaction between time and group F(1, 117) = 20.50, *p* < .001, ηp^2^ = .34 (Fig 3).

Post-hoc tests using the Bonferroni correction revealed Group A improved significantly between B1 and P1 (*p* < .001). There was then a significant improvement between P1 and B2 (*p* = .298) or between B2 and P2 (*p* = 1), showing that their performance improved after the intervention. For Group B, they improved significantly between B1 and P1 (*p* = .005) and between P1 and B2 (*p* = .030). After receiving the intervention, there was again a significant improvement in scores between B2 and P2 (*p* < .001).

Looking at the differences between the two groups at each time point, there was no significant difference between the two groups at B1 t(47) = 1.30, *p* = .200, *r* = .18 After Group A received the intervention, at P1, there was now a significant difference between the two groups t (48) = 8.35, *p* < .001, *r* = .77. This significant difference was maintained after the three week gap at B2 t(44) = 7.09, *p* < .001, *r* = .73. Then at P2, after Group B received the intervention, there was no longer any significant difference between the two groups t(44) = .07, *p* = .949, *r* = .01. This shows that there was a boost in performance for each group after completing the intervention, on top of any natural development. No differences were found with the two outliers (|*z*| > 2.5) excluded from analyses.

### CKAT battery

We then examined whether completing the intervention program had any far transfer effects to the CKAT using the same analysis structure.

**Fig 4:**
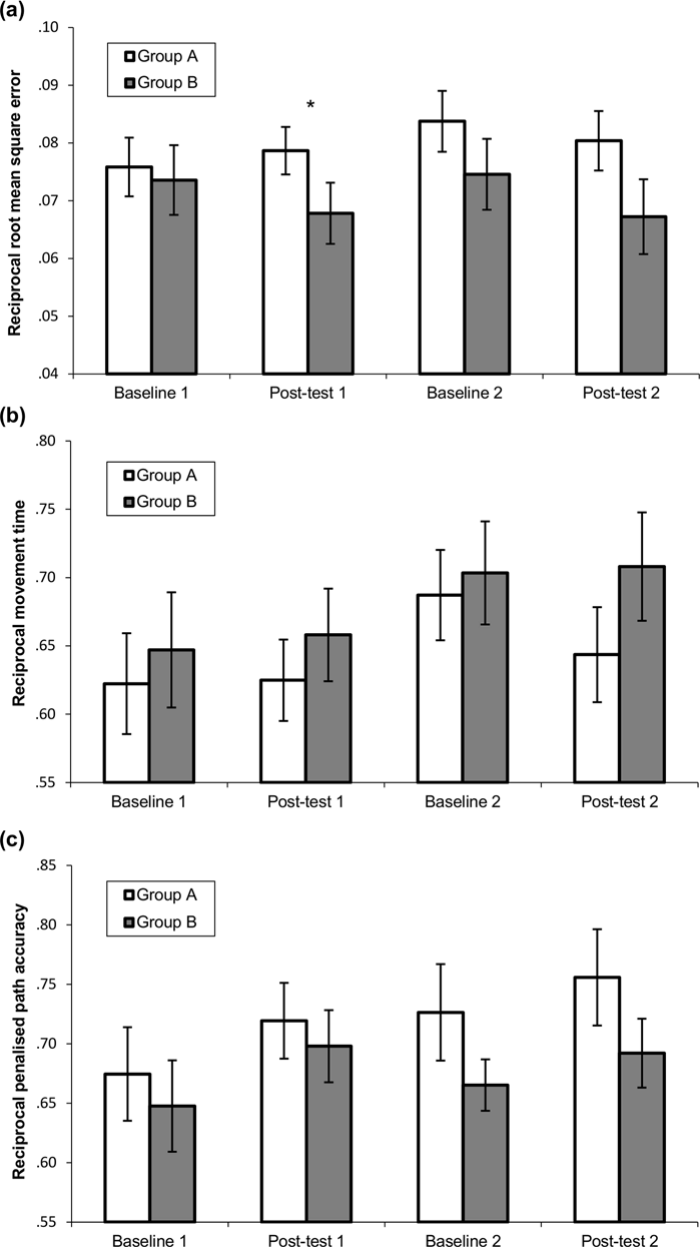
Performance on CKAT tracking, aiming and tracing tasks. *p < .05, **p < .001. Reciprocal scores with standard error for C-KAT variables: (a) tracking (root mean square error) (b) aiming (movement time) (c) tracing (penalised path accuracy)

For the tracking task (Fig 4A), there was no main effect of time F(3, 129) = .95, *p* = .418, ηp^2^ = .02, no main effect of group F(1, 43) = 2.16, *p* = .149, ηp^2^ = .05, or any interaction between time and group F(3,129) = .72, *p* = .543, ηp^2^ = .02, indicating that there was no overall improvement with or without the intervention on this specific task. This pattern of results was replicated with the two outliers for tracking removed.

For the aiming task (Fig 4B), there was a significant main effect of time F(3, 63) = 2.96, *p* = .039, ηp^2^ = .12, but no significant main effect of group F(1, 21) = .67, *p* = .422, ηp^2^ = .02, or interaction F(3, 63) = .40, *p* = .76, ηp^2^ = .019. However, using the Bonferroni correction, there were no significant differences between any of the time points when all of the children were grouped together and the main effect of time disappeared altogether when the three outliers were removed, indicating that there was no reliable improvement on the aiming task, with or without the intervention.

For the tracing task (Fig 4C), there was a significant main effect of time F(3, 135) = 2.86, *p* = .039, ηp^2^ = .06, but no significant main effect of group F(1, 45) = .82, *p* = .371, ηp^2^ = .02, or interaction F(3, 135) = .49, *p* = .687, ηp^2^ = .01. There were no significant differences between any of the time points when splitting the children by group (all *p* > .20), but as a whole sample, there were significant improvements between baseline 1 and post-test 1 (*p* = .034) and between baseline 1 and post-test 2 (*p* = .045), indicating that there was a general improvement over time on this task, irrespective of when the intervention was completed. There were no outliers for this task.

### Exploratory analysis and age feasibility

Previous work with this system has only used children over the age of 7 years, whereas children as young as 5 years were used in this study. The data were therefore split by school year into two groups: 5–7 years and 8–12 years. The same pattern or results for learning on the robotic arm was observed, with significant main effects and a significant interaction for both groups. The same pattern was also observed for the CKAT tasks, indicating that no particular age-group responded differently to the intervention.

Whilst all the children who took part in this study were classified as having manual coordination impairments by scoring under the 15^th^ percentile on the MABC, the MABC handbook suggests a score of under the 5^th^ percentile on the MABC-2 [24] should be used as a diagnostic threshold for Developmental Coordination Disorder, with those under the 15^th^ percentile (the threshold used in the current study) classed more broadly as being ‘at risk’. In addition, those children scoring ‘high’ or ‘low’ on the Beery Visual Perception task in a previous study [18] showed differing levels of improvement on the drawing task used in the study. Therefore, it was hypothesised that the intervention may have more or less of an effect depending on the initial level of severity of motor difficulties. The children were therefore split into two groups: those scoring under the 5^th^ percentile and those scoring over the 5^th^ percentile. No difference again were found for the pattern of results on the robotic arm novel task or the CKAT tasks between these groupings.

## Discussion

This study aimed to examine, using a counterbalanced-crossover design, whether the training benefits of an intervention delivered using a robotic-arm system would generalise to improvement on an objective assessment of manual coordination. We first confirmed through the use of a counterbalanced, crossover design that increases in performance on the novel task using the robotic arm system were directly attributable to the intervention, something that was not possible in previous studies examining this robotic system [17–20]. Group A (who completed the intervention in the first time period) showed significantly more improvement at the first post-test than Group B, who undertook no additional manual coordination training during this time. In addition, the crossover design showed that the improvement was maintained almost two months after the intervention was withdrawn. Some improvement was also observed in Group B over this first time period (before they took part in the intervention); this could be attributable to either natural development over time, or the fact that even performing the baseline and post-test using the equipment may have constituted ‘training’. However, the large difference in performance between the two groups was only eliminated after Group B had also completed the intervention in the second time period.

Secondly, we aimed to examine whether there was any evidence of the benefits generalising to the CKAT–an objective, computerised measure of manual ‘pen-skill’ ability. No evidence of improvement directly attributable to the intervention was found, with only the tracing task on the CKAT showing any improvement between time-points. It is always difficult to interpret a null-effect and part of the difficulty in establishing an effect may be due to the huge amount of variability in the data (consistent with the fact that all of these children had motor problems), which in a small sample may have masked any group-level effects. However, the current findings argue for increased caution when considering whether the intervention, as it currently stands, can be used to improve manual coordination generally.

Our initial hope was that a more generalised intervention might enable benefits to be extrapolated to a number of related tasks (for example, cutlery use and handwriting). If we had found evidence of generalisation to the C-KAT, it would give strong support for the use of this system as an intervention to improve ‘real-life’ tasks such as writing and cutlery use. It may be the case that it was some aspect of the CKAT tasks themselves which prevented generalisability of the training benefits being seen; whilst the CKAT tasks all involved ‘pen-skills’ (which we define as the ability to apply the appropriate forces to a handheld stylus so that a given visual output is achieved on a horizontally orientated plane) and compliance control, the CKAT tasks also involved skills such as prospective control and moving under forced time constraints, which may mask any improvements in the control mechanisms targeted by the robotic arm system. This also meant that different outcome variables had to be used for the robotic arm task and the CKAT tasks, something that again may have contributed to the lack of transfer observed. For example, the tracing task was arguably the most similar to the robotic arm task. However, the only way to progress on the robotic arm task was to move along the correct path, and therefore movement time was the only logical outcome variable whereas the tracing outcome measure needed to capture accuracy.

Alternatively, it may be that the actual tasks performed during the intervention need to be tailored to target specific skills such as handwriting. The robotic arm training involved moving in three dimensions which meant that movements involved the whole arm, as opposed to normal pen movement which involves mainly the wrist. There is evidence that learning similar skills with different effectors produces interference in generalisation [25], and therefore the training may also need to take place on a 2-D, horizontal plane. Likewise, the specific movement patterns involved in handwriting might need to be targeted for optimal intervention–for example, combining the type of repetitive letter writing used by Palsbo with their passive robotic system [26] with the active support and feedback of the current robotic device. Evidence from a meta-analysis by Smits-Engelsman and colleagues on the types of intervention for children with movement disorders would support the use of such a task-specific approach, and suggest this type of training may be important for generalisation [6].

The analysis did enable us to obtain evidence for the feasibility of using this type of system with a wider age range of participants than used in previous studies. An almost identical pattern of results was obtained for the children at all age bands and, most importantly, the training benefits on the robotic arm could be seen even in those aged as young as 5 years, which is ideal due to the emphasis on developing manual coordination skills such as handwriting at this age [27,28]. The system was readily deployed within the school environment, with the system (and outcome) being regarded positively by teachers and children. We should emphasise that the current study used researchers to supervise the intervention but there were no obvious reasons why teaching staff could not have undertaken this responsibility (and might even be better in this role given their experience of working with children from an educational perspective). However, once responsibility of implementing interventions such as this are handed over to the school, there are commonly problems with fidelity to the intervention schedule due to the other demands on the teachers and pupils during the school day. For example, in a feasibility study in schools using a different type of robotic intervention system to improve limb function in children with Cerebral Palsy [16,29], use of the system, designed to be used for half an hour per day, was actually used on average between 5 and 19 minutes per day, with technical problems often not being flagged by the staff. A greater evidence base of benefit would be required before the system could be recommended to schools as a feasible method of improving manual skills. Currently, the training relied on the use of existing robotic devices reprogrammed to run the intervention games, and therefore would be too expensive for mass distribution to schools. However, if the training regime could be altered to be of benefit for these children, then it would be possible to develop cheaper devices. It is worth noting that even the current price of these robotic systems makes delivery of this intervention (with multiple children receiving therapy at the same time) potentially far more cost effective than providing one-to-one specialised help.

In conclusion, this experiment has shown, using a strong methodological design, that the robotic arm system is effective at training manual skills in a wide age range of children with motor difficulties. However, no generalisation of benefit was found to a manual coordination assessment battery of pen-skills, indicating that it may be necessary for robotic systems to target task-specific actions in order to improve specific manual skills (such as handwriting).

## Supporting Information

S1 Dataset Dataset of participant’s responses on the (i) Robotic Arm novel task and (ii) CKAT outcome measures, at 1^st^ and 2^nd^ baseline and post-test assessments. This dataset also lists the following additional descriptive information for each participant: counterbalance group assignment, handedness, gender and age.
(XLSX)
